# Monoterpene Indole Alkaloids from the Aerial Parts of *Rhazya stricta* Induce Cytotoxicity and Apoptosis in Human Adenocarcinoma Cells

**DOI:** 10.3390/molecules27041422

**Published:** 2022-02-19

**Authors:** Zainab H. Abdul-Hameed, Nahed O. Bawakid, Hajer S. Alorfi, Tariq R. Sobahi, Najla Ali Alburae, Ahmed Abdel-Lateff, Serag Eldin I. Elbehairi, Mohammad Y. Alfaifi, Nabil A. Alhakamy, Walied M. Alarif

**Affiliations:** 1 Department of Chemistry, Faculty of Science, King Abdulaziz University, P.O. Box 80203, Jeddah 21589, Saudi Arabia; flowr9@hotmail.com (Z.H.A.-H.); nbawaked@kau.edu.sa (N.O.B.); halorfi@kau.edu.sa (H.S.A.); drtariq_s@hotmail.com (T.R.S.); 2 Department of Biology, Faculty of Science, King Abdulaziz University, P.O. Box 80203, Jeddah 21589, Saudi Arabia; nalbourai@kau.edu.sa; 3 Department of Pharmacognosy, Faculty of Pharmacy, Minia University, Minia 61519, Egypt; ahmedabdellateff@gmail.com; 4 Department of Natural Products and Alternative Medicine, Faculty of Pharmacy, King Abdulaziz University, P.O. Box 80260, Jeddah 21589, Saudi Arabia; 5 Department of Biology, Faculty of Science, King Khalid University, Abha 9004, Saudi Arabia; serag@kku.edu.sa (S.E.I.E.); alfaifi@kku.edu.sa (M.Y.A.); 6 Cell Culture Laboratory, Egyptian Organization for Biological Products and Vaccines, VACSERA Holding Company, Giza 22311, Egypt; 7 Department of Pharmaceutics, Faculty of Pharmacy, King Abdulaziz University, Jeddah 21589, Saudi Arabia; nalhakamy@kau.edu.sa; 8 Department of Marine Chemistry, Faculty of Marine Sciences, King Abdulaziz University, P.O. Box 80207, Jeddah 21589, Saudi Arabia

**Keywords:** Saudi plants, Apocynaceae, alkaloids, cytotoxicity, MCF-7, HepG2, HeLa

## Abstract

Chromatographic investigation of the aerial parts of the *Rhazya stricta* (Apocynaceae) resulted in the isolation of two new monoterpene indole alkaloids, 6-*nor*-antirhine-*N_1_*-methyl (**1**) and razyamide (**2**), along with six known compounds, eburenine (**3**), *epi*-rhazyaminine (**4**), rhazizine (**5**), 20-*epi*-sitsirikine (**6**), antirhine (**7**), and 16-*epi*-stemmadenine-*N*-oxide (**8**). The chemical structures were established by various spectroscopic experiments. Compounds **1**–**8** exhibited cytotoxic effects against three cancer cells with IC_50_ values ranging between 5.1 ± 0.10 and 93.2 ± 9.73 µM against MCF-7; 5.1 ± 0.28 and 290.2 ± 7.50 µM against HepG2, and 3.1 ± 0.17 and 55.7 ± 4.29 µM against HeLa cells. Compound **2** showed the most potent cytotoxic effect against all cancer cell lines (MCF-7, HepG2 and HeLa with IC_50_ values = 5.1 ± 0.10, 5.1 ± 0.28, and 3.1 ± 0.17 µM, respectively). Furthermore, compound **2** revealed a significant increase in the apoptotic cell population of MCF-7, HepG2, and HeLa cells, with 31.4 ± 0.2%, 29.2 ± 0.5%, and 34.9 ± 0.6%, respectively. Compound **2** decreased the percentage of the phagocytic pathway on HepG2 cells by 15.0 ± 0.1%. These findings can explain the antiproliferative effect of compound **2**.

## 1. Introduction

Cancer is an uncontrollable growth of cells, it could be generated or disseminated in different organs. It represents a major world health problem and increases the global mortality rate. Additionally, it is identified as the second cause of death after cardiovascular diseases [1]. In 2018, around 10 million people died from cancers [2]. The most common cancers were diagnosed in breast, lung, colon and rectum, prostate, skin (non-melanoma), and stomach with the number of cases, 2.26, 2.21, 1.93, 1.41, 1.20, and 1.09 million, whereas the most common causes of cancer death in 2020 were lung, colon, liver, rectum, stomach, and breast 1.80, 0.935, 0.830, 0.769, and 0.685 million according to GCO (Global Cancer Observatory) [1]. It continues to expand globally, exerting marvelous effects on governments and individuals. It causes negative effects on countries’ health systems, and has different effects on individuals, including physical, emotional, and financial strain [1,2].

Apocynaceae is a large family of flowering plants, consisting of 424 genera with more than 4600 species distributed in five subfamilies, Rauvolfioideae, Apocynoideae, Periplocoideae, Secamonoideae, and Asclepiadoideae. It could be trees, shrubs, woody vines, and herbs. Members of this Apocynaceae family are distributed primarily in tropical and subtropical areas of the world [3]. Many members of this family are poisonous and are used medicinally. These activities resulted from the presence of cardiac glycosides and various alkaloids including indole and steroidal alkaloids. Several genera having cytotoxic activity embrace *Catharanthus*, *Nerium*, *Plumeria*, *Tabernaemontana*, and *Ichnocarpus* [3,4,5].

Monoterpenoid indole alkaloids (MIAs) are an interesting class of naturally bioactive metabolites originating from the coupling of an indole-containing nucleus (i.e., tryptophan) and a monoterpenoid derivative (e.g., secologanin) [6,7]. MIAs are common compounds isolated from Apocynaceae, Rubiaceae, and Loganiaceae. Monoterpenoid indole alkaloids were reported to display anti-cancer, anti-inflammatory, analgesic, spasmolytic, insecticidal effects [8]. For example, ajmalicine and reserpine reported from the Apocynaceae plant, *Rauwolfia serpentina*, exhibited cardiopathy and antihypertensive activities. Additionally, camptothecin isolated from *Camptotheca acuminate* and vincristine and vinblastine isolated from *Catharanthus roseus* exhibited potent antitumor activity [9,10,11,12]. The dimeric molecule, vinblastine, with the commercial name Vinblastine^®^, is applied solely or in combination with other drugs for the treatment of choriocarcinoma and breast carcinoma besides their curation effect on Kaposi’s sarcoma, lymphomas, Hodgkin’s disease, and advanced carcinoma of the testis. The other dimeric monoterpenoid indoles, vincristine, which is marketed under the name, Oncovin, is used in combination with other anticancer drugs for the treatment of Hodgkin’s disease, lymphoma, acute leukemia, carcinoma, and sarcoma [13,14].

The genus *Rhazya* (Apocynaceae) is a rich source of monoterpenoid indole alkaloids. It includes two species, *Rhazya stricta* (*R. stricta*) and *Rhazya orientalis*. In the Arabian Peninsula, *R. stricta* is used in folk medicine for the treatment of several diseases [15,16,17]. Several MIAs as leepacine, aspidospermiose, aspidospermidose, strictanol, strictanine, and strictibine are terpenoidal were reported from *R. stricta*. Extensive studies on *R. stricta* alkaloids indicated their therapeutic effects as antitumor, antimicrobial, antifungal, and antihypertensive [17,18,19].

Conclusively, the current study focused on the separation of antiproliferative MIAs from the aerial parts of *R. stricta*.

## 2. Results and Discussion

### 2.1. Chemistry

**Figure 1:**
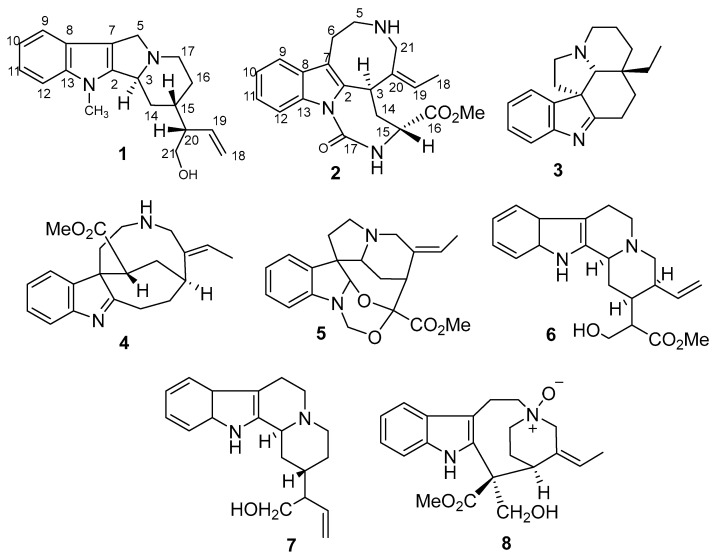
Compounds isolated from the aerial parts of *Rhazya stricta*.

Chromatographic separation of the CHCl_3_-EtOH (1:2) extract of the aerial parts of R. stricta yielded six MIAs, including a new antirhine derivative, 6-*nor*-antirhine-*N1*-methyl (**1**), together with five known compounds eburenine (**3**) [20], *epi*-rhazyaminine (**4**) [21], rhazizine (**5**) [22], and 20-*epi*-sitsirikine (**6**) [21] and antirhine **(7**); whereas the chromatographic separation of the acidified-water extract of the aerial parts of *R*. *stricta* yielded a new MIA, razyamide (**2**), along with one known MIAs, 16-*epi*-stemmadenine-*N*-oxide (**8**) [23] (Figure 1).

Compound **1** was obtained as a yellow oily substance of molecular formula C_19_H_24_N_2_O, as established by HR-EIS-MS. The presence of N-containing structure was evidenced from the orange color developed upon spraying with Dragendorff’s reagent; however, the presence of indole moiety has been evidenced from four absorption bands at 227, 276, 284, and 291 nm observed in the UV spectrum. Absorption bands at 3250, 3067, 1599, and 1456 cm^−1^ in the IR spectrum indicated the presence of OH, CH-, and a benzene ring, respectively. Four aromatic proton signals appeared doublets (d), doublets of doublets of doublets (ddd), doublets of doublets of doublets (ddd), and doublets (d) in the ^1^H NMR spectrum resonating at δ_H_ 7.38 (1H, d, *J* = 7.8 Hz), 6.96 (1H, ddd, *J* = 7.8, 7.8,1.2 Hz), 7.01 (1H, ddd, *J* = 7.8, 7.8, 1.2 Hz), and 7.30 (1H, d, *J* = 7.8 Hz), respectively, pointed out to the presence 1,2-disubstituted benzene ring (Table 1).

The ^1^H NMR spectrum indicated signals assigned to a terminal vinyl group resonating at δ_H_ 5.05 (1H, d, *J*= 1.8 Hz), 5.04 (1H, d, *J*= 1.8 Hz), and 5.69 (1H, ddd, *J*= 18.6, 12.0, 1.8 Hz), in addition to a *singlet* signal due to a quaternary methyl group resonating at 3.29 (3H, s). The ^13^C NMR and DEPT spectra suggested that compound **1** possessed 19 carbon signals, which was categorized into indole-ring signals [(δ_C_ 137.1 ppm (C, C-2), 107.5 (C, C-7), 128.3 (C, C-8), 118.3 (CH, C-9), 119.4 (CH, C-10), 121.4 (CH, C-11), 111.8 (CH, C-12), and 137.3 (C, C-13)], signals due to six methylenes, four methines, and one methyl carbons. Among them, two sp^3^ methylenes (δ_C_ 52.2 and 48.5 ppm), one sp^3^ methine (δ_C_ 55.6 ppm) attached to a nitrogen atom, and a methyl group (δ_C_ 49.6 ppm) linked to the indole *N*-atom.

The 2D spectra of compound **1** including the ^1^H-^1^H COSY, HSQC, and HMBC spectra showed great similarity to a previously isolated alkaloid known as antirhine) [24]. However, a deep comparison between both alkaloids revealed the absence of a methylene function in the case of **1** (CH_2_-6) and instead of the appearance of a methyl function. The location of the methyl function was deduced from the down-field absorptions in both ^1^H and ^13^C NMR and also from the correlation between these methyl protons and the carbons be linked to the nitrogen atom of the indole ring. From the comparison between the above results and literature data, compound **1** is greatly similar to antirhine [24], with some differences appearing when revising the HMBC spectrum with the correlation of the methyl group with C-2 and C-13. The relative stereochemistry of **1** was gleaned from the NOESY spectrum and by comparison of its chemical shift values with those of published data [24]. The cross-peak between H-15 and H-20 confirmed their cofacial orientation, and the absence of cross-peak between them and H-3 indicated the similarity of the stereochemistry of **1** with that of antirhine [24]. From the previous discussion, compound **1** and be identified as 6-*nor*-antirhine-*N*_1_-methyl (**1**) (Figure 1).

Compound **2** was obtained as a yellow oily substance of molecular formula C_20_H_23_N_3_O_3_, as established by HR-ESI-MS. The presence of *N*-containing structure was evidenced by the orange color developed upon spraying with Dragendorff’s reagent. Absorption bands at 3229, 2946, 1730, 1656, and 1450 cm^−1^ in the IR spectrum indicated the presence of OH, CH-, carbonyl ester, and benzene ring, respectively. Four aromatic proton signals appeared doublets (d), doublets of doublets (dd), doublets of doublets (dd), and doublets (d) in the ^1^H NMR spectrum resonating at δ_H_ 7.45 (1H, d, *J* = 7.6 Hz), 7.08 (1H, dd, *J* = 7.6, 7.6 Hz), 7.12 (1H, dd, *J* = 7.6, 7.6 Hz), and 7.28 (1H, d, *J* = 7.6 Hz), respectively, pointed out to the presence 1,2-disubstituted benzene ring (Table 2). The methyl group appeared δ_H_ 1.79 (3H) which was coupled to an olefinic H-atom at 5.38 (1H, q, *J*= 6.8), indicating an ethylidene side chain, and a singlet signal at δ_H_ 3.69, characteristic of a methyl ester group. The ^13^C NMR and DEPT spectra suggested that alkaloid **2** possessed 20 carbon signals, which was categorized into indole-ring signals [(δ_C_ 133.0 (s, C-2), 107.8 (s, C-7), 126.3 (s, C-8), 118.1 (d, C-9), 119.5 (d, C-10), 121.9 (d, C-11), 111.1 (d, C-12), and 136.6 (s, C-13)], along with signals assigned to four methylenes, three methines, two methyls, and three quaternary carbons (including two carbonyl carbons). The ^1^H-^1^H COSY spectrum established three aliphatic proton sequences H_2_-5-H_2_-6, H-3- H_2_-14-H-15 and isolated methylene protons H_2_-21. The presence of an indole ring (C-2, C-7, C-8 to C-13) was revealed by the HMBC correlations of the proton H-6 (δ_H_ 3.03) with C-2 and C-7, H-9, H-10, H-11 and H-12 to C-8 and H-9, H-10, H-11 to C-13 and H-9, H-12 to C-7. The HMBC correlations of H-5 and H-6 to C-7, H-6 to C-2, H-14 (δ_H_ 2.04) with C-2 (δ_C_ 133.0), C-15 (δ_C_ 53.7) and C-16 (δ_C_ 170.7) and the correlations of H-3, H_2_-14, and H-15 through COSY, all suggested the linkage C-2-CH-3-CH_2_-14-CH-15-C-16. The correlations between H_3_-18 (δ_H_ 1.79), H-19 (δ_H_ 5.38) and C-21(δ_H_ 3.91) establish the position of the ethylidene group and the attachments of C-20. The HMBC correlation of Me of ester (δ_H_ 3.69) and NH to C-16 indicated the connection of the ester and the NH to C-15. The NH appearing at (δ_H_ 7.92) indicated the amide group O=C-NH. The gross structure of **2** is illustrated in Figure 1. The NOESY spectrum of compound **2** exhibited no cross-peak between the H-3 and H-15, which implied their different orientation i.e one of them is α and the other is β-oriented. Compound **2** was identified as razyamide.

### 2.2. Biology

#### 2.2.1. Cytotoxicity

Compounds **1**–**8** exhibited cytotoxicity against three cancer cells with IC_50_ values ranging between 5.1 ± 0.10 and 93.2 ± 9.73 µM against MCF-7; 5.1 ± 0.28 and 290.2 ± 7.50 µM against HepG2 and 3.1 ± 0.17 and 55.7 ± 4.29 µM against HeLa cells (Table 3). Compound **2** showed the most potent cytotoxic effect against HepG2 with an IC_50_ value of 5.1 ± 0.28 µM, as well as on MCF-7 and HeLa cells with IC_50_ values = 5.1 ± 0.10 and 3.1 ± 0.17 µM, respectively. Compounds **4**, **6**, and **7** showed effective cytotoxicity on HeLa cells with IC_50_ values of 23.4 ± 2.07, 12.4 ± 1.51, and 23.2 ± 1.68 µM, respectively. However, compound **6** exhibited a cytotoxic effect against HepG2 cells with an IC_50_ value of 21.1 ± 1.97 µM. Compounds **3** and **8** exhibited a promising cytotoxicity effect towards all cancer cells (Table 3). Compounds **6** and **7** showed a similar cytotoxic effect against MCF-7 cells, whereas compound **7** showed cytotoxicity against HepG2. Moreover, compounds **4** and **5** showed the week cytotoxic effect against HepG2 with IC_50s_ of 290.2 ± 7.50 and 118.8 ± 8.48 µM, respectively.

#### 2.2.2. The Effect of Compound **2** on the Cell Cycle Distribution of Human Cancer Cells

**Figure 2:**
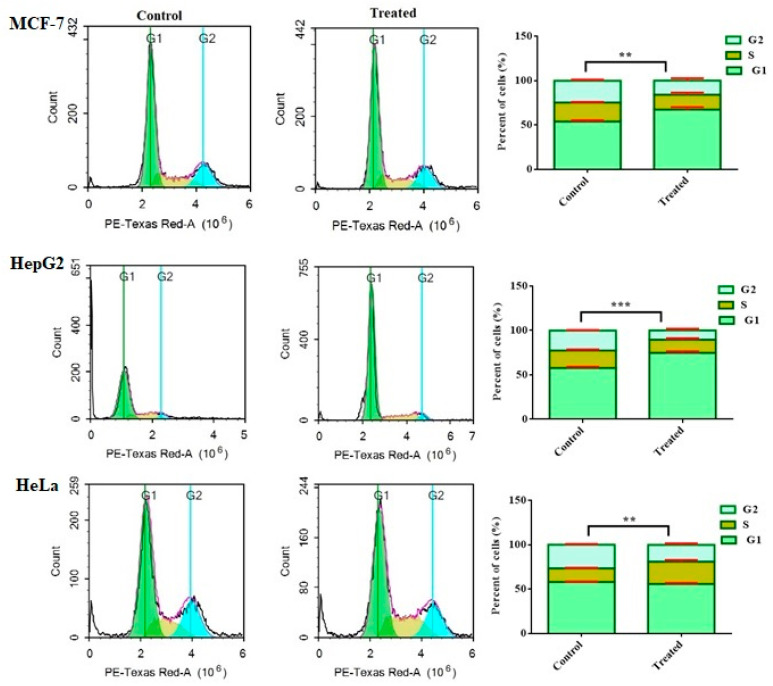
Effect of compound **2** on cell cycle phases of MCF-7, HepG2 and HeLa cells. Cell cycle distribution was determined using DNA cytometry analysis after exposure to **2** for 48 h. Data are presented as the mean ± SD; *n* = 3; ** *p* < 0.01 and *** *p* < 0.001.

Tracking cell cycle phases of the tumor cells were explored for anticancer effects. Therefore, the effect of compound **2** on the distribution of cell cycle phases was analyzed in MCF-7, HepG2, and HeLa cells using flow cytometry after treatment for 48 h. As illustrated in Figure 2, the proportion of MCF-7 and HepG2 cells in the G1 phase notably arrested increased by 25.7 ± 2.7% and 40.5 ± 2.0%, respectively, compared to the untreated cells. Meanwhile, the percentage of S phase in HeLa cells increased by 17.2 ± 1.8%. The percentage of cells in the G2/M phase significantly decreased by 59.2 ± 1.9% when HepG2 cells were treated with compound **2**.

#### 2.2.3. Assessing Cell Apoptosis with Annexin V-FITC

**Figure 3:**
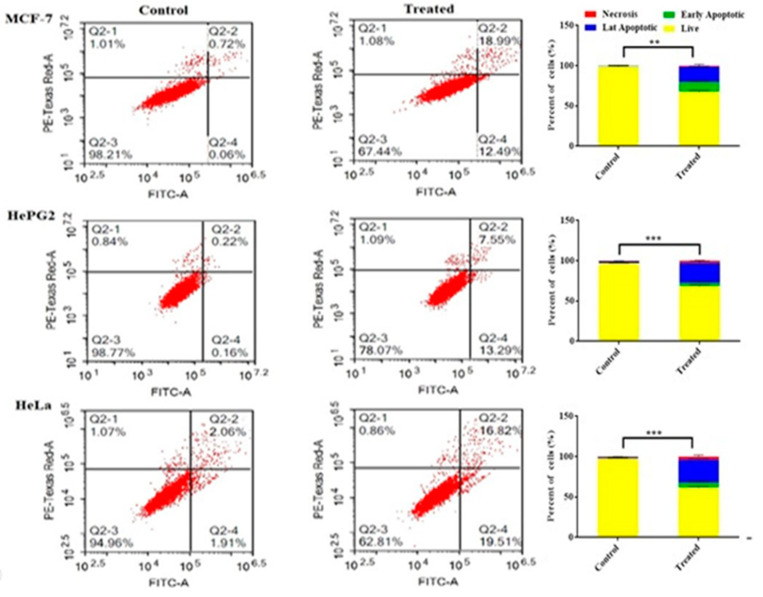
Apoptosis/necrosis assessment for compound **2** against MCF-7, HepG2, and HeLa cells subjected to previous treatment for 48 h, and apoptosis/necrosis quantified using flow cytometry. Data are presented as the mean ± SD; *n* = 3; ** *p* < 0.01 and *** *p* < 0.001.

For differential assessment of the cells undergoing apoptosis (programmed cell death) versus cells dying via necrosis (non-programmed cell death) in MCF-7, HepG2, and HeLa cells, annexin V-FITC/PI staining coupled with flow cytometer was performed (Figure 3). In the MCF-7, HepG2, and HeLa cells, a significant increase in apoptotic cell population was detected after treatment with **2**, with 31.4 ± 0.2%, 29.2 ± 0.5%, and 34.9 ± 0.6%, respectively, compared to the cell control. In comparison to the control, compound **2** demonstrated a significant rise in the necrotic cell population after treating cervix cancer cells (HeLa) followed by hepatocellular carcinoma cells (HepG2) with 3.7 ± 0.2% and 2.5 ± 1.1%, respectively.

#### 2.2.4. Assessment of Autophagy

**Figure 4:**
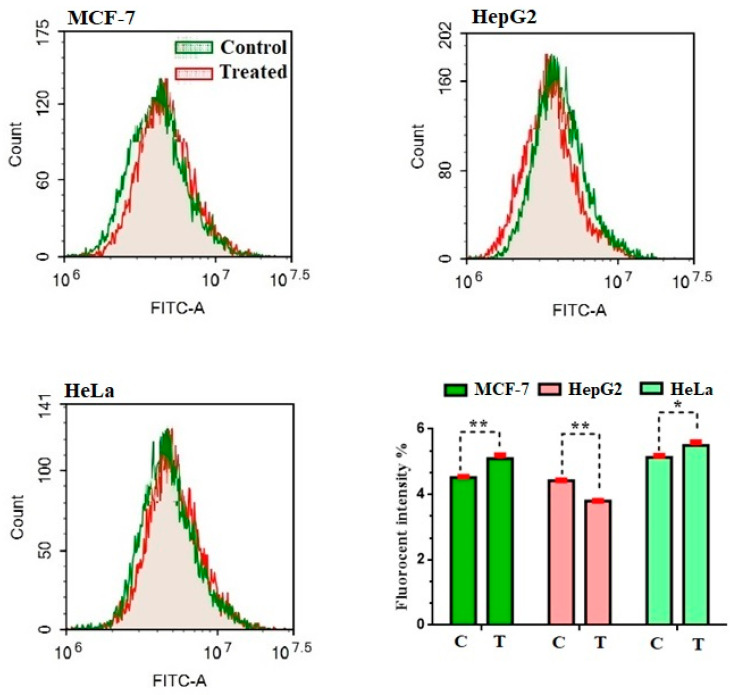
Effect of compound **2** on the autophagy cell death in MCF-7, HepG2, and HeLa cells. Exposure to treatments for 48 h. Data are presented as the mean ± SD; *n* = 3; * *p* < 0.05, ** *p* < 0.01.

Aside from apoptosis, autophagy-mediated programmed cell death is a hot topic in science. The effect of compound **2** on the autophagy process in MCF-7, HepG2, and HeLa cells was evaluated using Cyto-ID autophagy detection dye and flow cytometry (Figure 4). Autophagic cell death was increased, triggered in MCF-7 and HeLa cells by 13.3 ± 0.1% and 7.8 ± 0.1%, respectively, compared to the control percent. Additionally, the lowest percentage of the phagocytic pathway was observed with compound **2** on HepG2 cells by 15.0 ± 0.1% compared to the control.

## 3. Materials and Methods

### 3.1. General

Instrument specifications, solvent sources, and grades, chromatographic separation materials, and reagents are previously reported [21].

### 3.2. Plant Material

The plant material was identified and collected as previously reported [23].

### 3.3. Extraction and ISOLATION

The dried aerial parts of *R. stricta* (1 kg) were exhaustively extracted with a mixture of CHCl_3_: EtOH (1:2 *v/v*; 3 × 4 L; 25 °C) and yielded a dark residue (102.0 g). The residue was partitioned between 2% HCl and CHCl_3_ (1:1). The aqueous layer was separated, turned basic by wisely addition of ammonium hydroxide (pH = 10), and extracted with CHCl_3_. The CHCl_3_ extract was dried and yielded a basic material (20.0 g), which was directly chromatographed over aluminum oxide adsorbent (CC) and gradient elution from chloroform to ethyl acetate and then to methanol, to give five fractions (Fr. A–E). The fraction eluted by pure CHCl_3_ (Fr. A), was collected and dried. The TLC was monitored by spraying with Dragendorff’s reagent which stained the nitrogenous compounds with orange color. Fr. A was purified by PTLC employing pet. ether: CHCl_3_ (4:6, *v/v*) as a developing system, to yield **3** (2.4 mg, *R_f_*= 0.52) and semi pure **4**, which was purified by PTLC employing CHCl_3_: MeOH (9.5:0.5, *v/v*; **4**, 1.0 mg, *R_f_* = 0.41). Fr. B eluted by 5% CHCl_3_ in EtOAc, was purified using PTLC and CHCl_3_: MeOH (9.5:0.5) as eluent yielded **5** (0.8 mg, *R_f_* = 0.08). Fr. C, which was eluted by 20% EtOAc in CHCl_3_, was purified by PTLC using CHCl_3_: MeOH (9:1, *v/v*) yielded **6** (2.0 mg, *R_f_* = 0.75). Fr. D, which was eluted by 40% EtOAc in CHCl_3_, was purified by PTLC using CHCl_3_: MeOH (9:1, *v/v* and yielded **7** (1.2 mg, *R_f_* = 0.74). Fr. E, which was eluted by 50% EtOAc in CHCl_3_, was purified by PTLC using CHCl_3_: MeOH (9:1, *v/v*; **1**, 0.8 mg, *R_f_* value = 0.55). Another 1 kg of the aerial parts of *R. stricta* was extracted directly with acidified water and then was processed as previously reported [15]. The crude basic residue (15.0 g) was fractionated on an Al_2_O_3_ column, employing the gradient technique starting from CHCl_3_, to EtOAc, and then to MeOH. The fraction, which was eluted with 5% MeOH in CHCl_3_, was purified by PTLC using CHCl_3_: MeOH (8.8:1.2, *v/v*) to give **8** (1.2 mg, R*_f_* = 0.12). The fraction eluted with 15% MeOH in CHCl_3_ has been purified by PTLC using CHCl_3_: MeOH (84:16, *v/v*) to yield **2** (2.0 mg, R*f* = 0.73).

### 3.4. Chemical Characterization

#### 3.4.1. 6-*nor*-antirhine-*N1*-methy*l* (**1**)

Yellow oily substance; [α]_D_^22^ +50.3 (MeOH, 0.01); UV λ_max_ (MeOH) 227, 276, 284 and 291 nm; IR *ν*_max_ (CHCl_3_) 3250, 3067, 2924, 1599, and 1456 cm^−1^; ESI-MS (70 ev), *m*/*z* (rel.int.): 297.1 (100) [M+ H, C_19_H_25_N_2_O]^+^, 280.0 (20) [M^+^- OH], 266 (20) [M^+^- CH_2_OH], 153 (100) and 143 (80); HR-ESI-MS *m*/*z* 297.1960 [M+H]^+^(Calcd. for C_19_H_25_N_2_O, 297.1967); ^1^H and ^13^C NMR (C_3_D_6_O) (Table 1).

#### 3.4.2. Razyamide (**2**)

Yellow oily substance; [α]_D_^22^ −44.9 (CHCl_3_, 0.01); UV λ_max_ (MeOH) 227, 277 and 312 nm; IR ν_max_ (CHCl_3_) 3229, 2946, 1730, 1656, and 1450 cm^−1^; ESI-MS (350 ev), *m*/*z* (rel.int.): 353.1 (100) [M^+^, C_20_H_23_N_3_O_3_], 335.0 (20), 321 (70), 303 (22), 251 (100), 210 (25), 170 (45), 144 (50) and 108 (25); HR-ESI-MS *m*/*z* 354.1812 [M+H]^+^(Calcd. for C_20_H_24_N_3_O_3_, 297.1967);^1^H and ^13^C NMR (CDCl_3_:CD_3_OD) (Table 2).

### 3.5. Biological Activities

#### 3.5.1. Cell Culture

Human mammary gland, breast adenocarcinoma (MCF-7), hepatocellular carcinoma (HepG2), and human cervix adenocarcinoma (HeLa) cells were obtained from American Type Culture Collection (ATCC) and cultured in RPMI-1640 medium (Gibco, Thermo Fisher Scientific, Carlsbad, CA, USA). The culture media were complemented with 10% FBS (fetal bovine serum), and 100 units/mL PS (penicillin/streptomycin). The cells were incubated at 37 °C in a humidified atmosphere with 5 % CO_2_.

#### 3.5.2. Cell Viability Assay

MCF-7, HepG2, and HeLa cells were seeded in 96-well plates, approximately 2000 cells/well. Following 72 h of cell treatment with compounds **1**–**8**, the media was replaced with 150 µL of 10% TCA (trichloroacetic acid) (Sigma-Aldrich, St. Louis, MO, USA) for 1 h at 4 °C after washing with PBS1x, followed by 5 times washing with distilled water. Afterwards, 70 μL SRB solution (0.4 % *w/v*) (Sigma-Aldrich, St. Louis, MO, USA) was added for 10 min at room temperature in a dark place. Cells were washed with 1% acetic acid (Merck) three times and air-dried overnight. The protein-bound SRB stain was dissolved by adding 150 μL of 10 mM Tris Base (Merck) and the O.D. was measured at 540 nm using a microplate reader FluoStar Omega (BMG Labtec, Ortenberg, Germany) [25,26].

#### 3.5.3. Cell Cycle Analysis

MCF-7, HepG2, and HeLa cells were treated with the pre-calculated IC_50_ values of compound 2 for 48 h. Then, cells were harvested by trypsinization, twice washed with PBS (phosphate-buffered saline), fixed in ice-cold 60% ethanol at 40 °C, and re-washed in PBS. After that, cells were resuspended in 500 μL propidium iodide (PI) with RNase staining buffer, BD Pharmingen (Biosciences Inc, San Diego, CA, USA) and incubated for 30 min. Lastly, FACS analyses were executed utilizing the ACEA Novocyte™ flow cytometer, ACEA Biosciences Inc., San Diego, CA, USA. For every sample, data of 12,000 cells were assembled and distribution of cell cycle phases were analyzed applying ACEA Novo Express™ software, ACEA Biosciences Inc., San Diego, CA, USA [27]

#### 3.5.4. Apoptosis Analysis

MCF-7, HepG2, and HeLa cells were treated with compound 2 for 48 h, trypsinized, and washed twice with PBS. Apoptosis assessment was carried out via the Annexin V-FITC/PI Apoptosis Detection Kit, BD Biosciences, San Diego, USA, as stated by the manufacturer. In brief, cells were resuspended in 0.5 mL of binding buffer then 5 μL of Annexin V-FITC and 5μL of PI (staining solution) were added for 15 min at room temperature in a dark place. Finally, the cells were applied to FACS analysis using ACEA Novocyte™ flow cytometer, ACEA Biosciences Inc., San Diego, CA, USA, within one hour following staining, cell cycle distribution is calculated using ACEA. Novo Express™ software (ACEA Biosciences Inc., San Diego, CA, USA) [28].

#### 3.5.5. Autophagy Assay

Autophagic cell death was quantified using the Cyto-ID Autophagy Detection Kit (Abcam Inc., Cambridge Science Park, Cambridge, UK) to further explain the way by which cancer kills cells in response to compound 2 treatment. In brief, cells were treated to a predefined IC_50_ of 2, for 48 h while being exposed to a drug-free medium (control group). Cells were collected and washed twice with PBS after treatment. Cells were stained with Cyto-ID Green and incubated at 37 °C for 30 min in the dark, according to the manufacturer’s instructions. After staining, cells were injected and examined using ACEA Novocyte^TM^ flowcytometry (ACEA Biosciences Inc., San Diego, CA, USA) [28].

#### 3.5.6. Statistical Analysis

All data were analyzed using one-way analysis of variance (ANOVA.) Three replicates were used for each treatment. Differences between groups were considered significant at * *p* < 0.05, ** *p* < 0.01 and *** *p* < 0.001. Graphs were plotted using GraphPad Prism software, version 6.00 (GraphPad Software, La Jolla, CA, USA).

## 4. Conclusions

Two new monoterpene indole alkaloids (MIAs), 6-*nor*-antirhine-*N_1_*-methyl and razyzmide (**2)**, along with six known MIAs were isolated from the aerial parts of *Rhazya stricta*. The chemical structures were established using various spectroscopic measurements. Compounds **1**–**8** exhibited significant cytotoxicity against three cancer cells MCF-7, HepG2, and Hela. Compound **2** showed a potent antiproliferative effect against MCF-7, HepG2, and HeLa cells with IC_50_ values ≤ 5.1 µM. Additionally, it demonstrated a significant rise in the apoptotic cell population after treating MCF-7, HepG2, and HeLa with 31.4%, 29.2%, and 34.9%, respectively. Compound **2** decreased the percentage of phagocytic pathway on HepG2 cells by 15.0 *±* 0.1%. These findings can explain the antiproliferative effect of compound **2**.

## Figures and Tables

**Table 1:** ^1^H, ^13^C-NMR and HMBC of compound **1** ^a^.

Position	δ_C_ ^b^	δ_H_ ^c^	HMBC
2	137.1 s	-	11, 9
3	55.6 d	4.08 brs	19, 5a
5a 5b	52.7 t	3.16 dd (9.0, 4.8) 2.92 brs	
7	107.5 s	-	9, 5a
8	128.3 s	-	9, 10, 12
9	118.3 d	7.38 d (7.8)	11, 12
10	119.4 d	6.96 ddd (7.8, 7.8, 1.2)	12
11	121.4 d	7.01 ddd (7.8, 7.8, 1.2)	9
12	111.8 d	7.30 d (7.8)	10
13	137.3 s	-	9, 11, Me
14a 14b	32.1 t	2.14 m 2.10 m	
15	32.0 d	1.66 m	
16a 16b	28.6 t	1.70 ddd (16.8, 9.0, 4.2) 1.55 dd (9.0, 4.2)	Me
17a	48.5 t	2.77 m	
17b		2.68 m	
18a 18b	116.8 t	5.05 d (1.8) 5.04 d (1.8)	
19	140.5 d	5.69 ddd (18.6, 12.0, 9.6)	18a, 18b, 21a, 21b
20	49.6 d	2.28 brs	18a, 18b, 19
21a 21b	63.9 t	3.66 dd (10.8, 6.0) 3.62 dd (10.8, 6.0)	19
N-CH_3_	49.6 s	3.29	

^a^All assignments are based on 1D and 2D measurements (HMBC, HSQC, COESY). ^b^ Implied multiplicities were determined by DEPT (C = s, CH = d, CH_2_ = t). ^c^ *J* in Hz.

**Table 2:** ^1^H, ^13^C-NMR, and HMBC of compound **2** ^a^.

Position	δ_C_ ^b^	δ_H_ ^c^	HMBC
2	133.0 s	-	
3	27.7 d	4.45 d (7.6)	7
5a 5b	50.3 t	3.17 dd (11.0,4.5) 2.65 brs	6,7,21
6a 6b	20.3 t	3.03 m 2.78 d (17.0)	2, 7
7	107.8 s	-	
8	126.3 s	-	
9	118.1 d	7.45 d (7.6)	7,8,11,12,13
10	119.5 d	7.08 dd (7.6,7.6)	8,9,11,12,13
11	121.9 d	7.12 dd (7.6,7.6)	8,9,12,13,2
12	111.1 d	7.28 d (7.6)	7,8,10
13	136.6 s	-	
14a 14b	33.6 t	2.64 br.s 2.04 ddd (16.1,10.3,9.3)	2,7,16, OMe
15	53.7 d	3.82 brs	16, OMe
16	170.7 s	-	
17	168.4 s	-	
18	13.1 q	1.79 brs	19,20
19	121.9 d	5.38 q (6.8)	3,18,20,21
20	133.0 s	-	
21a 21b	59.0 t	3.91 d (13.6) 3.14 brs	6,7,15,20
OCH_3_	51.2 t	3.69 s	16,7
NH	-	7.92 s	3,7,16,20

^a^ All assignments are based on 1D and 2D measurements (HMBC, HSQC, COESY). ^b^ Implied multiplicities were determined by DEPT (C = s, CH = d, CH_2_ = t). ^c^ *J* in Hz.

**Table 3:** Cytotoxic effects of compounds **1**–**8** isolated from *Rhazya stricta*.

Compound No.	IC_50_ (µM)		
	MCF-7	HepG2	HeLa
1	68.9 ± 3.45	52.7 * ± 2.02	30.7 * ^†^ ± 2.67
2	5.1 ± 0.10	5.1 ± 0.28	3.1 * ^†^ ± 0.17
3	50.7 ± 2.29	40.0 * ± 3.40	55.7 * ^†^ ± 4.29
4	40.5 ± 1.89	290.2 * ± 7.50	23.4 * ^†^ ± 2.07
5	93.2 ± 9.73	118.8 * ± 8.48	36.9 * ^†^ ± 2.18
6	52.2 ± 2.60	21.1 * ± 1.97	12.4 * ^†^ ± 1.51
7	65.4 ± 3.67	60.7 ± 3.30	23.2 * ^†^ ± 1.68
8	50.0 ± 2.31	54.9 ± 2.00	32.8 * ^†^ ± 2. 70
Doxorubicin	1.96 ± 0.02	1.8 ± 0.01	3.1 * ^†^ ± 0.15

Human mammary gland, breast adenocarcinoma (MCF-7), hepatocellular carcinoma (HepG2), and human cervix adenocarcinoma (HeLa). Data are presented as the mean ± SD; *n* = 3. * Significantly different from corresponding MCF-7 value, ^†^ Significantly different from corresponding HepG2 value.

## Data Availability

Data are available from the authors.

## Sample Availability

Samples of the compounds are available from the authors.
